# Switching It up with New KRAS Inhibitors

**DOI:** 10.1021/acs.jmedchem.5c00998

**Published:** 2025-04-22

**Authors:** Alex G. Waterson

**Affiliations:** † Departments of Pharmacology and Chemistry, Vanderbilt University School of Medicine, 2220 Pierce Avenue, Nashville, Tennessee 37232, United States

## Abstract

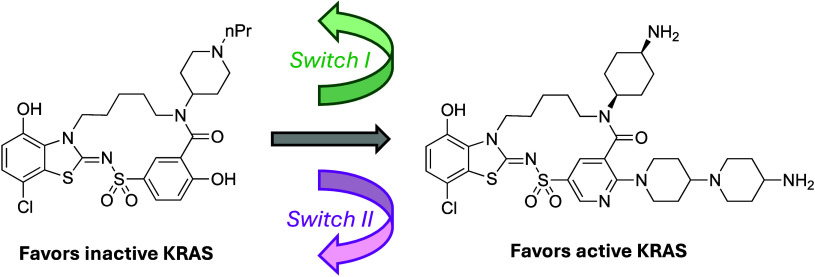

Alongside significant recent advancements in drugging an historically notorious oncogene, scientists continue to uncover new ways to target KRAS. This viewpoint summarizes the newly reported discovery of a novel chemical template that features the ability to tailor inhibition to different activation states of nucleotide-bound KRAS from the same scaffold.

“RAS is undruggable!” is no longer the mantra that it used to be, with two molecules that target the KRAS G12C mutation approved by the FDA for use in some nonsmall cell lung cancers and more agents that target this and other oncogenic mutants currently under clinical investigation.^1^ In a Featured Article(2) in this issue of *J. Med. Chem.*, a multi-institutional team of authors from Cancer Research Horizons—a part of CRUK, Scotland—as well as Novartis and the National Cancer Institute’s RAS initiative, discloses another intriguing way to target RAS mutants. This new work centers on compounds that bind to the Switch I/II pocket, with activity that can be tailored to the inactive or active forms of KRAS, and uniquely features a heretofore unreported “interswitch” subpocket.

Sotarasib and Adagrasib represent breakthroughs in the RAS field, standing as the first approved therapies that act directly upon KRAS.^1^ As will likely be familiar to many readers, these compounds bind in a pocket underneath the Switch II region on KRAS and covalently interact with the cysteine of the oncogenic G12C mutation, a strategy first described in ground-breaking work by Ostrem, Shokat, and colleagues.^3^ That initial discovery of electrophilic small molecule fragments that react with KRAS cysteine 12 and occupy the Switch II pocket provided a direct line-of-sight to the now-approved compounds. Switch II binders can also access other mutant forms of KRAS with proper elaboration. For example, compounds similar to Adagrasib can be made to interact with KRAS G12D via an electrostatic interaction.^4^ Further, a fragment screen using a modified form of RAS^5^ revealed reversibly binding compounds that occupy the Switch II pocket, and elaboration of these compounds can lead to binding to multiple KRAS isoforms.^6,7^

Another exceptionally promising approach has been reported by Revolution Medicines, who have used proximity-induced pharmacology to create clinical stage RAS inhibitors such as RMC-6236 that address several RAS mutants.^8^ In contrast to the reported Switch II pocket binders, this work focused on the active “RAS(ON)” GTP-bound conformation of KRAS, sequestering it into a complex with Cyclophilin A and thus sterically preventing the binding of KRAS effectors.

The identification of compounds that bind at the Switch I/II pocket and can also block the binding of RAS with other proteins was an important early part of the modern resurgence in RAS inhibitors.^9,10^ While compounds at this site can bind the active conformation of RAS, advanced inhibitors that utilize the Switch I/II pocket are rarer. In one example, reversible RAS binding under 1 μM has been achieved by virtue of a sophisticated structure-based design campaign.^11^ The newly reported work falls into this theme, upping the ante by discovering low nanomolar binding molecules.

Like many medicinal chemistry campaigns, particularly against difficult targets, the authors in this Featured Article took a broad initial perspective. Combining elements of analogs of literature-reported compounds such as **1** in Scheme 1 with fragment hits such as **2** from their own screening campaign led to a viable lead **3**. Initial SAR surveys around this lead allowed for the determination of structural information that clarified the binding modes of the compounds in the KRAS Switch I/II pocket. Using the structural information to reengineer the hit series to hydrogen bond with Asp54 and Glu37 and to pick up a halogen bond with Tyr71 led to compound **4**, which was determined by NMR titration to bind to GDP-bound KRASG12D with 20 μM affinity. The NMR-derived affinity for this compound against the active form of KRASG12D using a nonhydrolyzable analog of GTP (GNP) was found to be 6-fold weaker. Noting the potential for building in additional pocket interactions with nearby residue Asp38, the team grafted amine-containing extensions onto the phenolic sulfonamide and then enforced the protein-bound conformation of the molecule via macrocyclization, producing significant affinity gains. Indeed, compound **5** was determined to bind to GDP•KRASG12D with <50 nM affinity, as assessed by SPR, setting a new standard for a molecule that binds to the KRAS Switch I/II pocket. SPR also revealed that, like other compounds in the series, the affinity to the active form of the protein was weaker.

**Scheme 1:**
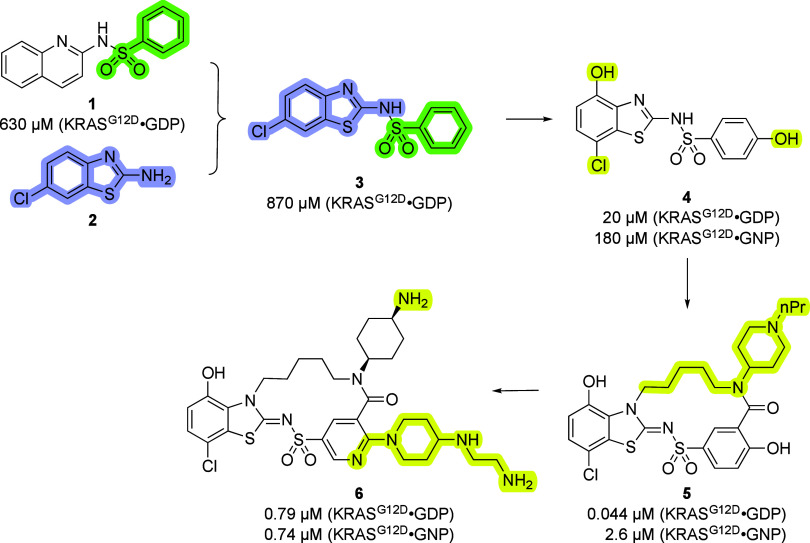
SAR Progression of RAS Inhibitors Key molecular changes that drove affinity advancements are highlighted.

A unique twist to this paper led to improved binding to the active conformation of KRAS. Inspired by the famously dynamic nature of the Switch regions,^12^ the team explored the extension of these compounds toward often-mutated G12, creating molecules such as **6** that feature submicromolar affinity to both the active and inactive KRASG12D conformations. Multiple crystal structures of this subclass of inhibitors bound to the protein reveal that the analogs can induce a rotation in the protein that swings Glu37 out of the way (see Figure 1), opening a channel in the protein surface that is dubbed by the authors as the “interswitch” pocket. Interestingly, compounds that access this pocket can also switch the binding preferences. For example, **7**, which does not fully access the new pocket, displays higher affinity to GDP-bound KRASG12D, while interswitch-binding **8** inverts that preference, ranking among the most potent published Switch I/II pocket binders to the active form of KRAS. Intriguingly, the close proximity of these interswitch binders to the terminal phosphate of the nucleotide suggests a future opportunity for an additional interaction.

**Figure 1:**
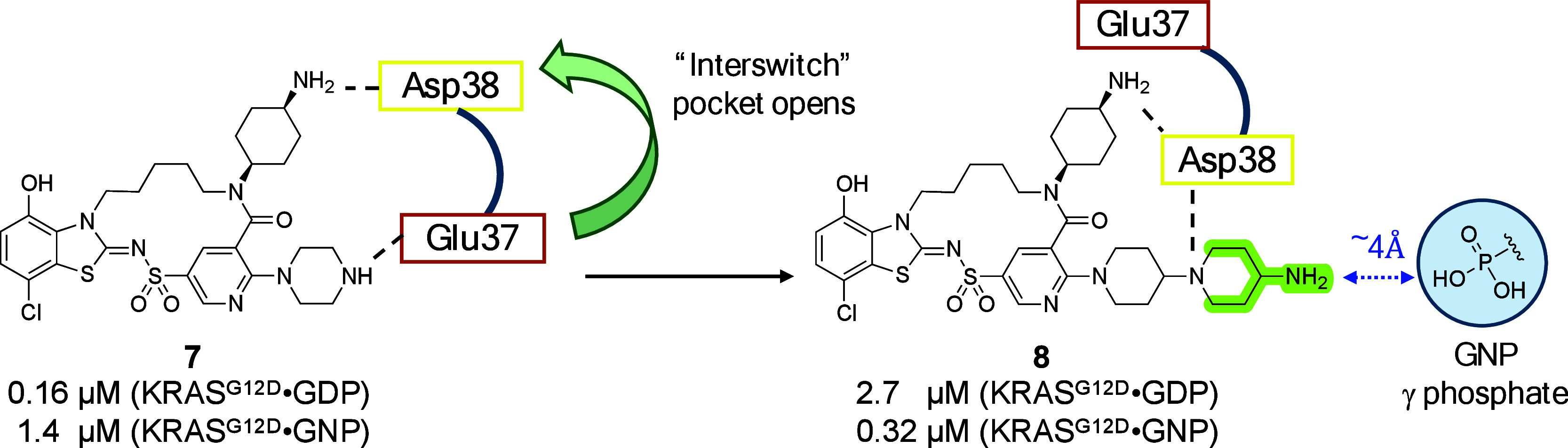
A conformational shift opens the interswitch pocket.

In just over a decade spanning the identification of some of the first structurally characterized modern RAS binders^3,9,10^ to the first approved KRAS drugs,^1^ scientists across the globe have steadily chipped away at the façade of what had long been considered one of the toughest, yet most important, target opportunities in oncology-focused drug discovery. As the field matures, the patterns of clinical efficacy and the mechanisms of resistance to the earliest drugs are being revealed. To improve patient outcomes, we will increasingly turn to complementary options—KRAS inhibitors that effectively target additional oncogenic mutations will surely be needed, as will drugs that bind to multiple pockets on the protein and that operate by different mechanisms of action.

The compounds newly reported in this feature article bind to the Switch I/II pocket and can demonstrate the ability to engage KRAS and modulate the MAPK pathway in cancer cells. However, due to a mixed *in vitro* ADME profile, they do not yet represent potent *in vivo* tools or clinically relevant options. Nonetheless, the rare tunability of the template, allowing for multivariant binding to several mutant forms of RAS and allowing access to both the inactive GDP-bound and active GTP-bound conformations of the protein, represents an attractive launching point for the discovery of new and more advanced inhibitors at the Switch I/II pocket. Further, the first report of the interswitch pocket raises an intriguing new avenue for further elaboration and improvements for these compounds and other reported analogs. It will be exciting to monitor the future of this work!
